# Stroke Ready: a multi-level program that combines implementation science and community-based participatory research approaches to increase acute stroke treatment: protocol for a stepped wedge trial

**DOI:** 10.1186/s13012-019-0869-3

**Published:** 2019-03-07

**Authors:** Lesli E. Skolarus, Anne E. Sales, Marc A. Zimmerman, Casey L. Corches, Zach Landis-Lewis, Maria Cielito Robles, A. Camille McBride, Narmeen Rehman, Alina Oliver, Nishat Islam, Mellanie V. Springer, Alison O’Brien, Sarah Bailey, Lewis B. Morgenstern, William J. Meurer, James F. Burke

**Affiliations:** 1 Stroke Program, University of Michigan Medical School, 1500 E Medical Center Dr, Ann Arbor, MI 48109 USA; 2 Department of Learning Health Sciences, University of Michigan, 1111 E. Catherine St, Ann Arbor, MI 48109 USA; 3 VA HSR&D Center for Clinical Management Research, VA Ann Arbor Healthcare System, 2215 Fuller Rd, Ann Arbor, MI 48105 USA; 4 School of Public Health, University of Michigan, 1415 Washington Heights, Ann Arbor, MI 48109 USA; 5 Bethlehem Temple Church, 3401 M L King Ave, Flint, MI 48505 USA; 6 Bridges into the Future, Flint, MI 48507 USA; 7 Emergency Department, University of Michigan Medical School, 1500 E Medical Center Dr, Ann Arbor, MI 48109 USA; 8 Department of Neurology, VA Ann Arbor Healthcare System, Ann Arbor, MI 48105 USA

**Keywords:** Implementation science, Community-based participatory research, Health behavior theory, Tailored implementation in chronic disease, Acute stroke, African Americans

## Abstract

**Background:**

Post-stroke disability is common, costly, and projected to increase. Acute stroke treatments can substantially reduce post-stroke disability, but few patients take advantage of these cost-effective treatments. Practical, cost-efficient, and sustainable interventions to address underutilized acute stroke treatments are currently lacking. In this context, we present the Stroke Ready project, a stepped wedge design, multi-level intervention that combines implementation science and community-based participatory research approaches to increase acute stroke treatments in the predominately African American community of Flint, Michigan, USA.

**Methods:**

Guided by the Tailored Implementation of Chronic Disease (TICD) framework, we begin with optimization of acute stroke care in emergency departments, with particular attention given to our safety-net hospital partners. Then, we move to a community-wide, multi-faceted, stroke preparedness intervention, with workshops led by peer educators, over 2 years. Measures of engagement of the safety-net hospital and the feasibility and sustainability of the implementation strategy as well as community intervention reach, dose delivered, and satisfaction will be collected. The primary outcome is acute stroke treatment rates, which includes both intravenous tissue plasminogen activator, and endovascular treatment. The co-secondary outcomes are intravenous tissue plasminogen activator treatment rates and the proportion of stroke patients who arrive by ambulance.

**Discussion:**

If successful, Stroke Ready will increase acute stroke treatment rates through emergency department and community level interventions. The stepped wedge design and process evaluation will provide insight into how Stroke Ready works and where it might work best. By exploring the relative effectiveness of the emergency department optimization and the community intervention, we will inform hospitals and communities as they determine how best to use their resources to optimize acute stroke care.

**Trial registration:**

ClinicalTrials.gov Trial Identifier NCT03645590.

**Electronic supplementary material:**

The online version of this article (10.1186/s13012-019-0869-3) contains supplementary material, which is available to authorized users.

## Background

Post-stroke disability is common, costly, and projected to increase [1]. Acute stroke treatments reduce the relative risk of post-stroke disability by over 30%, yet despite their benefits and cost-effectiveness, they are administered to less than 5% of stroke patients in the USA [2–6]. The mainstay of acute stroke treatment is an intravenous (IV) medication, tissue plasminogen activator (tPA), and more recently endovascular treatment [7]. These acute stroke treatments are both rigidly time limited and sensitive. They must be administered in the emergency department (ED) within hours of the onset of the stroke—earlier treatment results in a much greater chance of stroke recovery (i.e., time is brain) [8].

Both the pre-hospital and hospital environment contribute to acute stroke treatment underutilization [9–11]. Pre-hospital delays are the largest contributor to acute stroke treatment underutilization [10]. Calling emergency services for stroke is associated with decreased pre-hospital delays, yet only about 50% of stroke patients arrive at the hospital via ambulance [12–17]. In addition to pre-hospital delays, hospitals also contribute to low acute stroke treatment rates [18]. When stroke patients arrive at the hospital, a multistep process occurs to determine whether the patient is eligible for acute stroke treatments. Hospitals vary widely in their abilities to execute these complex treatment pathways [18]. Pre-hospital and hospital delays reduce the likelihood and efficacy of treatment, as each minute delay results in an estimated loss of 1.9 million neurons [19].

### Racial differences in stroke

African Americans suffer disproportionate stroke incidence, prevalence, and post-stroke disability [20–22]. African American stroke patients are less likely to call emergency services, have greater pre-hospital delays, and even if eligible are less likely to receive acute stroke treatments than white stroke patients in the USA [18, 23, 24]. These inequities in post-stroke disability can be at least partially addressed with interventions to increase acute stroke treatment rates. To address the underutilization of acute stroke treatments, particularly among African Americans, we designed the Stroke Ready project, a multi-level project to increase acute stroke treatments in the predominately African Americans community of Flint, Michigan.

## Methods

### Overview

**Fig. 1:**
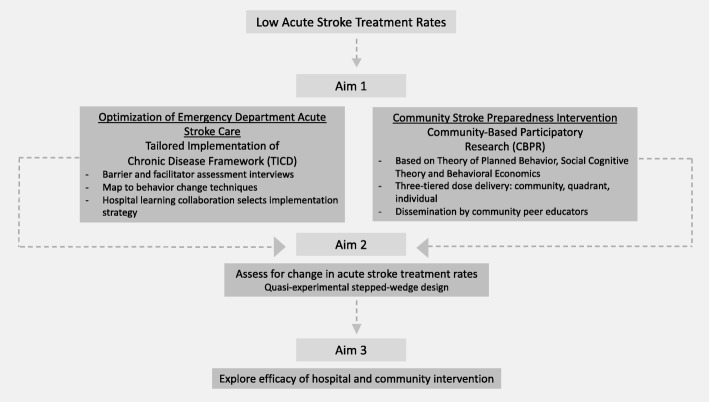
Stroke Ready program overview

The Stroke Ready project is a quasi-experimental, multi-level intervention that combines implementation science and community-based participatory research approaches (Fig. 1). We begin grounded in implementation science to optimize acute stroke care in Flint EDs, with particular attention given to safety-net hospital partners. Then, through a community-based participatory research approach, we move to a health behavior theory-based, multi-faceted, peer educator-led, community-wide stroke preparedness intervention. We have the following specific aims.

Specific aim 1: To adapt and expand our community-based participatory research developed, theory-based, Stroke Ready pilot community intervention and implement a hospital-based intervention to optimize acute stroke care in an urban safety-net, hospital.

Specific aim 2: To increase acute stroke treatment rates in Flint, Michigan through a two-pronged approach of hospital and community level interventions.

Hypothesis 1: The socio-ecologically motivated, theory-based, culturally sensitive Stroke Ready program, that includes both hospital and community elements, will increase acute stroke treatment rates (primary outcome).

Specific aim 3: To inform future community-based participatory research (CBPR) interventions by exploring both the relative importance of community and hospital interventions and the efficacy of the intervention on processes mediating the outcome.

### Location of the Stroke Ready program

Flint, Michigan, USA the birthplace of the automobile company General Motors, was once a thriving industrial city. Like many cities in the industrial US Midwest, the collapse of the automobile industry exacerbated the economic struggles of the city [25]. Present day, 60% of the population is African American and over 40% live below the poverty level [26]. Furthermore, Flint is experiencing a water crisis due to high lead levels in the drinking water, heightening health concerns in the community [27]. Flint has (1) high rates of stroke [28] and (2) low acute stroke treatment rates [6]. In the USA, a mean of 4.2% of ischemic stroke patients receive acute stroke treatment, but in Flint, the mean treatment rate is 2.2% [6]. In addition, acute stroke ED care is not optimized. Stroke Ready, motivated by this acute stroke practice gap, will strive to increase acute stroke treatment rates in Flint, Michigan.

### Community-based participatory research

Community-based participatory research (CBPR) is a collaborative approach to research where the community is fully engaged with academic partners and both share the responsibility of conceiving, designing, testing, and disseminating interventions to improve the health of the community [29]. Our partnership was established in 2009 and is composed of academic partners from the University of Michigan, including vascular neurologists and experts in health behavior and health education, and community partners from faith and community-based organizations. To ensure broad community representation, we also established a community advisory board (CAB). The CAB, co-chaired by the academic and community principal investigators, suggests strategies for recruitment, facilitates relationships between the research team and other organizations, recommends approaches for community intervention delivery, and most importantly promotes sustainability of the Stroke Ready program.

### Study procedures

#### Stroke Ready emergency department acute stroke care optimization

The goal of implementation research is to promote the uptake of research findings or best practices into clinical practice or policy with a particular focus on healthcare personnel and organizational behavior [30]. Before increasing the number of acute stroke patients who present to the ED, it is important that ED acute stroke care is optimized. Our primary focus is at a low-performing safety net hospital. At the time of grant submission, this hospital had below average quality measures and did not participate in US national stroke quality improvement initiatives. Additionally, the majority of stroke patients cared for at the safety-net hospital are African American, suggesting an opportunity to improve health equity.

Improving processes can improve the quality of care [31]. Acute stroke treatment is a multistep process with a goal of administration of acute stroke treatment within 60 min of the stroke patient arriving to the ED—faster times suggest more optimized processes [32]. We use the plan-do-study-act (PDSA) process framework to guide our implementation process. [33, 34]. To begin, a hospital learning collaborative, including key hospital stakeholders hospital administrators, EMS personnel, physicians, nurses and technicians, will be established [35]. We will then assess the barriers and facilitators of guideline concordant acute stroke treatment guided by the tailored implementation in chronic disease (TICD) framework. The TICD explores determinants of practice in seven domains: guideline factors, health professional factors, patient factors, professional interactions, incentives and resources, capacity for organizational change, and social, political, and legal factors. The TICD guides the development of a semi-structured interview guide and qualitative analysis. The determinants identified in the semi-structured interviews of acute stroke providers are mapped to behavior change techniques, whereby a multifaceted implementation strategy will likely occur [36–38]. Data is collected on the results of the implementation strategy and the clinical outcome then fed back to the hospital learning collaborative to make changes as needed. Finally, the TICD-based interview guide and any created implementation strategies will be made available for other safety-net hospitals to optimize acute stroke care.

### Stroke Ready community intervention

#### Pilot program and health behavior theory

**Fig. 2:**
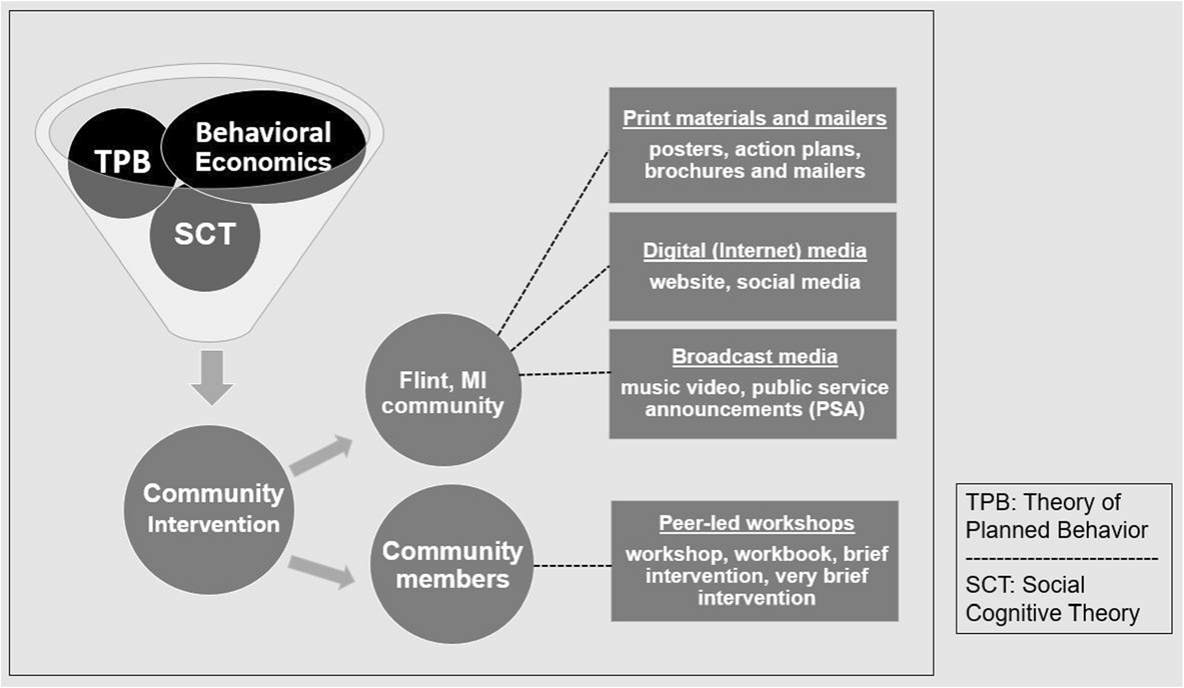
Overview of Stroke Ready community intervention health behavioral conceptual model and components

The Stroke Ready community intervention conceptual model is largely based on the Theory of Planned Behavior with some elements from Social Cognitive Theory and Behavioral Economics, as indicated in Fig. 2. According to the Theory of Planned Behavior, the biggest, most immediate predictor of behavior change is the intention to change the behavior [39]. Behavioral intention is a function of three psychological constructs: attitude toward the behavior, subjective norms (the perceived social pressure to perform the behavior), and perceived behavioral control (the perception of ease or difficulty with which one can perform the behavior). We also placed emphasis on aspects of the Social Cognitive Theory, focusing especially on self-efficacy (an individual’s belief in their ability to perform specific behaviors). We previously identified the psychological constructs of attitude toward the behavior, social norms, and self-efficacy to call 911 as problematic in Flint [39–41]. Consistent with Theory of Planned Behavior and Social Cognitive Theory, Stroke Ready will focus on these constructs to increase behavioral intent to call 911 and ultimately increase 911 calls and decrease ED arrival time in an acute stroke situation. In addition to health behavior theory, we incorporated insights from behavioral economics, namely pre-commitment and behavioral nudge. Research shows that when participants commit to performing a certain behavior, the likelihood that the individual will engage in the designated behavior increases. By completing Stroke Ready action plans in a public forum, Stroke Ready aims to increase behavioral intent to call emergency services and shift social norms [42, 43]. Behavioral nudges serve as a way to alter choice architecture, or default responses, and can be effective when stressing a simple response (such as, calling emergency services) to a complex problem [44, 45]. Participants will be educated in the workshops and then, through a series of nudges, the behavior of calling emergency services will be continually reinforced by mass media and encouragement of the action plan to be placed in a position of prominence in their homes.

#### Stepped wedge design: Stroke Ready quadrant delivery

**Fig. 3:**
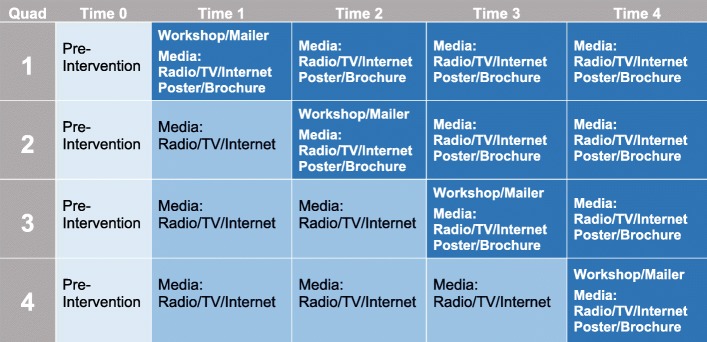
Stroke Ready quadrant dissemination plan

Stroke Ready begins with the ED intervention and then moves to the community intervention. The Stroke Ready community intervention will be delivered in four quadrants defined by the community’s geographic boundaries (Fig. 3). By the end of the 2-year period, 6 months in each quadrant, all components of the intervention will have reached the entire community. At the end of 6 months, the materials will remain in the quadrant, but the research teams’ focus will shift to the next quadrant. Though delivery in the active quadrant is preferred, given our commitment to CBPR, we would not forego a workshop, if requested, in a quadrant that is not currently active. The stepped wedge design will (1) allow more efficient use of resources, (2) optimize intervention efficacy by increasing the likelihood of repetitive exposure to the intervention, and (3) allow for exploratory analyses to determine whether the hospital or community intervention is most important and clarify the efficacy of the overall intervention.

### Community intervention composition

The Stroke Ready community intervention contains four components (Fig. 2) that work together to reinforce messaging of stroke preparedness. Stroke Ready is committed to reaching the entire community. While many community members attend community events and workshops, others may be less engaged. Thus in addition to peer educator-led activities, the Stroke Ready program also includes print media, broadcast media, and social media.

#### Peer-led workshops and brief sessions

Stroke Ready is committed to building community capacity and ensuring stroke preparedness sustainability [46]. Therefore, we will train a local workforce, peer-educators, who will deliver the Stroke Ready information to the community. Peer-educators will be hired from within the community and local colleges. They will undergo 6–8 h of training and a confirmation of their readiness to lead a Stroke Ready workshop will be assessed by the Stroke Ready research staff. The training includes education on Stroke Ready workshop materials, strategies for public presentation, and facilitation skills to aide peer educators in creating an open environment for learning.

We developed four versions, of various durations (5, 15, 30, and 60 min), of the workshop to accommodate various settings. To safeguard program integrity, we established core workshop components to ensure quality and consistency of program messaging across workshops. These core components include stroke definition, impact of stroke, stroke is an emergency, stroke is treatable, stroke signs (FAST), timing is everything: call 911, and join the 100,000’s of Americans who have been treated.

The very brief session, which lasts about 5 min, is a one-on-one discussion between a peer educator and community member. This session type was designed for concise delivery of stroke-preparedness education at events, such as health fairs and festivals, using the Stroke Ready brochure and the stroke action plan card. The second option is a 15-min, peer educator-led, brief session using the brochure and stroke action plan card as primary means of delivery, but includes select peer-led interactive activities (e.g., guided group discussion and self-learning assessments) not included in the very brief session.

We also have a 30-min workshop, which was designed specifically in response to requests from local businesses to have an option able to be delivered over their employee lunch hours. The 30-min workshop is facilitated by the peer educator and includes a workbook, providing Flint-tailored stroke preparedness education, as well as interactive activities (e.g., role play, group discussions, and self-learning assessments). Finally, in addition to the stroke-preparedness workbook and interactive activities, the 60-min workshop includes a PowerPoint presentation (or a flip/chart easel) and audio recording to facilitate content delivery, allowing for educational information to be delivered at a more moderate pace. Participants may be given nominal gifts, such as pens with the Stroke Ready logo and silicone bracelets. Our goal is to have 75 workshops/sessions delivered throughout the 6-month time period per quadrant (× 4 quadrants).

#### Print materials and mailers

##### Print material

The Stroke Ready program will include a print media campaign with posters, action plans, workbooks, mailers, and brochures. Feedback from our community focus groups ensures that print materials are culturally sensitive and include accessible language.

##### Posters

To expand reach and extend exposure of messaging to those who may not receive mailers or choose to attend a workshop, three posters were created, each with a distinct primary message: “don't be silent about stroke,” “act FAST if you see signs of stroke,” and “time is everything” (Additional file 1: Figure S1). Each poster also uses graphics, large fonts, and bright colors to catch attention and make the messaging easily comprehensible for individuals on the move. The posters will be disseminated by the research team in various highly frequented businesses and locations around the quadrant of focus as well as city busses.

##### Mailers

A community-wide mailer will be sent to every residential address in Flint [47]. The mailer will include a Stroke Ready information letter, brochure, magnet, and action plan. The magnet enables recipients to post their action plan on the refrigerator or any other viewable magnetic surface. Mailers will be sent out in each quadrant at the beginning of that quadrant’s 6-month period.

##### Workbooks

Workbooks to accompany the content of the 30- and 60-min workshops were created. These workbooks include all core components mentioned previously, activities held within the workshops, additional stroke preparedness information, a certificate of completion, a short quiz, and an action plan (discussed below). Workshop participants keep the workbooks.

##### Action plan

The action plan that is included within all Stroke Ready workbooks, in Stroke Ready mailers, and as an insert card with the brochures (provided during brief and very brief sessions) has been developed to encourage behavioral intent to call 911 when signs of stroke are observed. This action plan is formatted like a pledge that participants fill out with their own information, promising to themselves that they will remain “Stroke Ready.” The reverse side of the action plan includes the specific steps to follow after calling 911 for mental rehearsal of the behavior, as well as to increase ease of performance during an actual event. We encourage the action plan be put in an area of public display, such as on the refrigerator, as their commitment to calling 911, and also as a strategy to increase the effectiveness of the pre-commitment [48, 49].

##### Brochure

Brochures were developed to maximize dissemination of Stroke Ready messaging by condensing all Stroke Ready core components into a succinct, easily digestible format. These brochures will be used for stroke-preparedness education in brief and very brief sessions and will also be included in the mailers.

#### Digital (internet) media—website and social media

##### Website

The Stroke Ready website (https://www.strokeready.com/) will serve as a central repository for Stroke Ready information relating to the Stroke Ready campaign and workshops, information about stroke and resources, printable versions of brochures and flyer-size versions of posters, links to community partner sites, and events in the Flint community. The link will be placed on print materials and peer leaders will be asked to disseminate the Stroke Ready website address via their email, Facebook, and text messaging contacts.

##### Music video

The music video was developed during the Stroke Ready pilot by a local music director, and produced and sung by local musicians. It incorporates the FAST stroke symptoms mnemonic (e.g., F-face drooping, A-arm weakness, S-speech difficulty, T-time to call 911) into an original gospel-based music score and video [50]. Portions of the video were adapted to include community members acting out signs of stroke and of spreading the word about stroke symptoms, while maintaining its strong focus on self-efficacy in asking viewers to participate in demonstrating stroke signs [40]. The Stroke Ready music video will be available on the Stroke Ready website, Facebook page, and may be played during the workshops pending media capabilities.

##### Social media

Social media channels include a Facebook page and Instagram account. The Facebook page will include posts with details on upcoming workshops, community events, or stroke-related facts. Original content such as photos or videos from past community events and outreach will also be featured on the Stroke Ready Facebook and Instagram page. The hashtag “#StrokeReady” is included on posts to promote the Stroke Ready campaign and to invoke interest in our social media accounts. Social media accounts are developed and overseen by members of the research team and community.

#### Broadcast media—radio public service announcements

A 60-s version of the song from the music video was created for use as radio public service announcements (PSAs). Two additional PSAs, one to improve stroke awareness and the other focused on outcome expectations, will be delivered. These will be played on three local radio stations—a gospel station, a talk-radio station, and an adult contemporary station—each of which were chosen based on popularity within the Flint community in order to reach the most diverse listening audience possible. PSAs will play with greater frequency when the intervention launches into a new quadrant in hopes of building community interest in Stroke Ready. Frequency of plays will then decrease over the remaining months to serve as reinforcement of the messaging while other intervention components are ramping up.

### Participant recruitment

The Stroke Ready workshops are the only component for which the research team will actively recruit. The research team and Stroke Ready peer educators will work together to recruit organizations and participants for the Stroke Ready workshops through announcements, flyers, internet platforms, and word-of-mouth. To make the workshops as accessible to as many people as possible, workshops will be held in convenient locations and times (e.g., after church services, during lunch breaks). Additional factors that facilitate program recruitment are (1) the workshops are free and provide stroke education, (2) recruitment materials and intervention components were created by the community, for the community, (3) our CBPR approach helped us gain support of local leadership and community members with strong ties in the Flint community, (4) children will be allowed to attend as materials were developed and tested with youth input as well, and (5) workshops include audio portions derived from written scripts in the participant workbooks. This assures inclusion of both hearing and vision impaired participants.

#### Inclusion/exclusion criteria

Workshops will be open to the public. While our primary outcome measure is stroke treatment rates in Flint, stroke preparedness is a public health message applicable to most people; therefore, participants will not be excluded if they live outside the city limits. Similarly, the workshops are designed for English-speaking adults; however, non-English speaking individuals will not be excluded. Print materials will be translated into Spanish by one of the peer educators. Additionally, the Michigan School for the Deaf is located in Flint; therefore, the educational information for workshops will also be interpreted for the deaf and hard of hearing community.

### Outcomes

#### Primary and secondary outcomes

The primary outcome is acute stroke treatment rates, which includes intravenous tissue plasminogen activator (IV tPA) (MS-DRG 61–63 or ICD-9 procedure code 99.10), endovascular treatment (MS-DRG 21–23 or CPT codes 37184–6, 37201, 75896), and the combination [6]. Secondary outcomes are IV tPA treatment rates and the proportion of stroke patients who arrive by ambulance. These outcomes will be obtained from the electronic medical record and billing data of the three hospitals in Flint, which together account for 95% of all stroke treatments in Flint residents. For the primary and secondary outcome of acute stroke treatment rates, the study population will be patients with a primary diagnosis of ischemic stroke [51, 52]. For the secondary outcome of arrival by ambulance, the population will include stroke patients who present to the ED.

#### Tertiary/exploratory outcomes: emergency department

The tertiary/exploratory outcomes include arrival time (i.e., stroke symptom onset to ED arrival) and ED treatment time (i.e., ED arrival to acute stroke treatment) which will be obtained from the local hospital’s Get with the Guidelines Data. Get with Guidelines (GWTG) Stroke is a quality improvement registry sponsored by the American Heart Association and used by hospitals across the USA, including the three hospitals in Flint. Given the timeframe and quality of GWTG Stroke data in the three hospitals, we conservatively decided these would be exploratory outcomes.

#### Tertiary/exploratory outcomes: community

The community survey, Speak to Your Health (STYH), is a biennial, geo-coded survey that has been designed and administered by the Flint community, Genesee County Health Department since 2003 [53]. This survey assesses stroke attitude, self-efficacy, social norms, and stroke preparedness at the community level. It was administered in 2015 and 2017, continuing into 2019 and possibly 2021. In 2019 and 2021, we will add stroke education exposure questions to the STYH surveys. Additionally, these same questions will be added to the Flint Area Study (FASt), a longitudinal cohort study of Flint residents which includes face to face interviews with Flint residents. These community level assessments will assess community level change in the core components of the community intervention and community exposure to Stroke Ready.

### Process evaluation

Implementation and process data will be collected. The implementation measures for the hospital intervention will assess the engagement of the local hospital and the feasibility and sustainability of the implementation strategy. Engagement will be measured by frequency and attendance at hospital learning collaborative meetings. Feasibility and sustainability will be assessed by whether implementation strategies are implemented and sustained after the initial engagement period. The community intervention will broadly include measures of intervention reach, dose delivered, and satisfaction that will be assessed across multiple levels of the community intervention: community-level, quadrant-level, and individual-level (Additional file 2: Table S1).

#### Community-level

Those community intervention components that remain in circulation throughout the entire community of Flint for the duration of the 2 years, by nature of their respective formats (e.g., radio and TV PSAs, digital, and social media), will be measured at the community level. To determine the proportion of reach for Stroke Ready music video, website, and social media account page views coming from viewers within the Flint community, we will identify which views came from internet protocol (IP) addresses within Flint. Descriptive data will be collected to measure dose delivered, such as number of PSA plays on radio/TV, and number of views for website, music video, and social media pages. TV and radio stations will provide estimates of number of viewing/listening audience from the Flint community to determine reach for PSAs. Dose received for all digital and social media will measure participant satisfaction and engagement with content, such as “views,” “liking,” “following,” or “sharing.”

#### Quadrant-level

The intervention methods that are launched by quadrant (e.g., mailers, posters, workshops, and brief/very brief interventions) will be tracked and counted, with a summation of results at the end of the 6-month period for each quadrant. The measures for dosage delivered will be number of mailers sent and number of posters hung. Dosage received for mailers will be measured by tracking number of community members who mention seeing or receiving a mailer. Recruitment will be measured by number of sites where posters are displayed, and sites that have hosted workshops, brief, or very brief session in the given quadrant, as well as number of workshops/brief/very brief sessions held, by type.

#### Individual-level

Peer educators will collect the number of participants (reach), number of materials distributed, workshop/brief/very brief session duration, and content delivered (dosage delivered). Field-level observations including how participants are experiencing the program (responsiveness and satisfaction), as well as setting appropriateness for intervention delivery will be used to inform the intervention’s strengths and weaknesses, provide context for what components may, or may not be working, and any lessons learned. These data will provide the research team the opportunity to address issues or make improvements to the intervention delivery in real-time.

Individual-level intervention fidelity will also be assessed by the research team on a regular basis to ensure that the intervention types are being implemented as designed and that there is consistency in manner of delivery across peer educators. During these fidelity assessments, peer educators will be assessed using an observation form designed to measure adherence to intervention length, content, methods, and activities, facilitation quality, and participant responsiveness. Notes will also be taken during this time to record any environmental aspects that may influence intervention implementation or study outcomes. Peer educators will be provided immediate feedback about their performance including areas of strength, suggestions for improvement, and, if necessary, any required additional training.

### Outcome analyses

The primary analysis will be an interrupted time series comparison of acute stroke treatment rates in the three Flint hospitals. The pre-intervention period will be defined as at least 36 months prior to the start of the rollout of the hospital intervention. The intervention period will start with the implementation of the hospital intervention and continue through the complete rollout of the community intervention (45 months). All patients admitted with a primary diagnosis of ischemic stroke in both the pre-intervention and intervention periods will be included in the analysis. Logistic regression will be used to estimate the overall intervention efficacy (indicator variable) in a model predicting receipt of acute stroke treatment (binary variable). If a temporal trend exists in the pre-intervention period, we will adjust for the month since the start of the pre-period as a fixed effect while accounting for clustering at the hospital level. To maximize statistical power, the entire intervention (hospital and all community quadrants) will be parameterized with a single variable. With this approach, statistical power for the primary analysis will be more than adequate. Using hospital administrative and Medicare data, we estimate at least 480 strokes per year will occur at the 3 Flint hospitals for a total of at least 1440 strokes in the pre-period and 1800 in the post-period. Assuming a doubling in treatment rates (pre-intervention Medicare treatment rate 2.2%) [6], we will have over 90% power to detect this difference considering a two-sample binomial difference in proportions. This estimate is consistent with prior simulation work based on ARIMA analyses (effect size of 1.0) (pre-intervention monthly treatment rate = 2.2%, standard deviation = 2.1, predicted post-intervention treatment rate 4.3%, auto-correlation = 0.3) [54]. An effective doubling in treatment rates is a realistic, and possibly even conservative assumption, given that prior community interventions to increase acute stroke treatment rates increase treatment rates by 2.6 times. Furthermore, as the hospital intervention *Target Stroke* also approximately doubled acute treatment rates [32], it is highly plausible that a combination of our community and hospital interventions will lead to a doubling in treatment rates.

#### Secondary analyses: regional comparisons and quadrant-based analyses to enhance causal inference

As with any pre-post intervention design, the primary analysis is susceptible to confounders that may influence treatment rates and occur concomitantly with the intervention. Secondary analyses will explore the extent that such confounding may influence the primary analysis and enhance the ability to draw causal inferences from the primary analysis. First, we will repeat the primary analysis with a concurrent control group consisting of other large Michigan metropolitan regions (regional control model) where African Americans make up more than 25% of the population (Detroit, Saginaw, Muskegon, Benton Harbor). This analysis will control for regional effects that may lead to increased treatment rates that occur simultaneously with our intervention in Flint using data from the Michigan State Inpatient Database (SID), which collects data on all acute care hospitalizations in the state of Michigan within a given year. Second, by delivering the intervention sequentially to geographic quadrants within Flint, we will explore whether increases in acute treatment rates parallel the geographic pattern of intervention roll out (geographic model). Specifically, each stroke patient in Flint will be geocoded to one of the four intervention quadrants using EMR data and the Google geocoding interface. Our primary analysis will then be repeated by modifying the intervention indicator variable to represent whether the intervention was active in the patient’s geographic quadrant at the time of intervention. This analysis was not chosen as the primary analysis because of concerns about the potential for cross-quadrant contamination and because this approach leads to a modest reduction in statistical power.

#### Exploratory analyses: efficacy of program components and temporal patterns to inform future interventions

To inform future interventions, we will also perform a series of hypothesis-generating analyses to explore which elements of the program were most effective and the temporal properties of the program. Due to power concerns, our primary analysis does not consider the difference between the hospital and community effects. Thus, we will first estimate the proportion of the change in the acute stroke treatment rate attributable to the hospital-based intervention vs. the community-based intervention by repeating our geographic model including an indicator variable representing the time period of the hospital intervention as well as a community interaction term. In this way, we will be able to explore whether the Stroke Ready hospital or community-based intervention was more efficacious and whether there was synergy between the interventions. The stepped wedge design is a key innovation to this end. In typical multi-level interventions when all of the elements are rolled out nearly simultaneously, it is impossible to estimate which elements have the highest leverage; however, with the stepped wedge design, it is possible to gain a greater understanding of which elements are most important. Using simulation analyses, we estimate there will be 70% power to find a doubling at the community level, 55% power to find a doubling at the hospital level, and 21% power to find a doubling through a community-hospital interaction. Because this power is inadequate for a hypothesis-testing evaluation, we have specified this analysis as an exploratory analysis of which the purpose is to enhance our understanding of the importance of intervention elements and to inform future interventions. Second, we will determine the temporal properties of the Stroke Ready intervention by adding a linear slope term and exploring quadratic terms in our geographic model to estimate the time delay between intervention and changes in treatment rates and whether treatment rates level off or decline as the intervention persists into its later years. Finally, a strength of our data collection approach is that we will be able to inexpensively assess the sustainability of the intervention effect using Michigan SID data years after the intervention is completed without needing to perform additional data collection. Together, such analyses will determine the sustainability of the intervention and inform future interventions.

#### Analyzing secondary outcomes and process measures

The secondary outcome of tPA only treatment rates will also be assessed in an interrupted time series comparison of acute stroke treatment rates in the three Flint hospitals. Changes in the proportion of patients arriving by ambulance over time will be assessed using logistic regression with an indicator variable representing the intervention period. Changes in the time from ED presentation to acute stroke treatment will be explored using linear regression with a similar indicator variable representing the intervention period. Pre-post participant surveys will be compared with multi-level ordinal logistic regression (Likert-based outcomes) or logistic regression (binary outcomes) with a random participant-level intercept. Stroke preparedness and behavioral constructs will be assessed with ordinal logistic regression (Likert-based outcomes) or chi-squared tests (binary outcomes) with indicator variables representing the survey wave. Process measures will be summarized with descriptive statistics, as pre-intervention values will be either unmeasurable or unintelligible, formal statistical comparisons will not be performed.

### Cost-effective analysis

The research team will assess the cost-effectiveness of the overall Stroke Ready program. Cost-effectiveness will be estimated for two intervention scenarios: Stroke Ready delivery and Stroke Ready development and delivery. This will inform the value of taking the Stroke Ready intervention “out of the box” and delivering it in a novel context and to separately assess the cost of developing and delivering a similar intervention in a novel context.

Cost inputs to the models will be carefully recorded throughout the project. All Stroke Ready material expenditures (e.g., development, print media production, website maintenance) will be tracked, and as appropriate, assigned to either the hospital or community portion of the intervention. By summing work time costs and material costs, we will be able to estimate the total costs of the overall intervention and the hospital and community interventions separately. We will then separately estimate total quality adjusted life years (QALYs) gained by the Stroke Ready program (and separately for the hospital and community interventions) by applying the primary outcome treatment effect size to the total hospitalized population (i.e., 2.2% increase in treatment rates * 500 strokes = 11 additional patients treated) and estimated QALY gain using published stroke cost-effectiveness models [55, 56]. Estimated hospital and community effect sizes will be obtained from our secondary analysis assessing intervention component efficacy. The age distribution of patients who receive treatment via the intervention will be obtained from the overall Flint stroke population. By using repeated bootstrap samples from this population and repeatedly running the model, we will estimate 95% credible intervals on the QALY gain. We will then estimate the incremental cost-effectiveness ratio (ICER) by dividing estimated costs / estimated QALY gain across all scenarios.

## Discussion

The Stroke Ready project is a quasi-experimental, multi-level intervention that combines implementation science and community-based participatory research approaches to increase stroke treatment rates in an underserved, predominately African American community. If successful, the Stroke Ready program will provide a strategy to improve acute stroke treatments in safety-net hospitals and a community intervention to increase stroke preparedness that is easy to deliver, allowing for sustainability. An important goal of the Stroke Ready project is to quantify the effect size and the cost of ED optimization compared to the community intervention. These results will assist communities, hospitals, and perhaps even insurers in prioritizing either ED acute stroke readiness or community stroke preparedness when working to increase acute stroke treatment rates with limited resources.

The optimal outcome to assess the effectiveness of the hospital and community interventions separately is limited by the quality of available data. The measure that would most likely best reflect the effect of the community intervention would be arrival time (i.e., stroke onset to ED arrival). While this outcome is available in Get with the Guidelines Stroke registry, the time of stroke onset variable is missing in about 50% of patients, and there is high variability between hospitals making it an inadequate measure for Stroke Ready. For these reasons, arrival time is an exploratory outcome in Stroke Ready. Given the associations of calling 911 with increased acute stroke treatment rates, our second choice for a community intervention outcome would be EMS arrivals. We anticipate abstracting this variable from the hospital billing data or the electronic medical record. While imperfect, due to EMS availability and community concerns, such as lack of trust, we believe EMS arrivals is our best option to assess pre-hospital delay. Regarding the hospital intervention, the ultimate outcome is ED treatment time (i.e., ED arrival to acute stroke treatment time) and the proportion of eligible ischemic stroke patients who receive acute stroke treatment. ED treatment time will be an exploratory outcome while we will not assess treatment among eligible patients due to concerns about data quality, particularly in the extended time window, and the duration of data capture from the hospitals. Future studies could consider deliberative data capture of arrival time, hospital time, and the proportion of eligible ischemic stroke patients who receive acute stroke treatment, but this would dramatically increase the resources required to assess the effectiveness of stroke preparedness programs.

We believe the Stroke Ready Program will be sustainable through several mechanisms: (1) developed and tested with a CBPR approach so that the community has ownership of Stroke Ready and ensuring that it is culturally and locally relevant, (2) training of peer educators who will have knowledge of stroke warning signs and the importance of calling 911, (3) a complete community intervention package that can be administered with little to no training, (4) well-positioned CAB to promote sustainability, and (5) optimized acute stroke care in a safety net hospital. Future studies could be performed to assess the sustainability of the Stroke Ready Program through these mechanisms.

The Stroke Ready program has some limitations. It was designed to reflect the local Flint culture, a strength in this program, but may require adaptation for dissemination to other communities with different populations. The Stroke Ready program has multiple components, but of relatively low intensity. Thus, while our design will not permit us to differentiate which aspect of the community intervention contributes to changes we expect to observe, it will provide us with information about how such a multi-faceted CBPR approach may be an effective strategy to address stroke disparities. In addition, the low intensity of the components of our multi-factorial approach is somewhat low cost and easy to implement in its entirety thereby not unduly burdening the dissemination of the program to other communities. Our decision to deliver workshops outside of the designated quadrant if requested by the community weakens the study design as there will be some cross over among the quadrants. However, we believe that our commitment to CBPR and to our ultimate goal of increasing acute stroke treatment rates in the Flint community supersedes this limitation. Finally, by promoting increased usage of 911 for stroke, Stroke Ready may increase 911 calls and ED visits for non-stroke and non-emergencies. Given that acute stroke treatment is cost-saving, assessing this societal trade-off could be considered.

In summation, if successful, the Stroke Ready program will directly benefit the Flint community by decreasing post-stroke disability. Furthermore, it will inform future acute stroke interventions in underserved, predominately African American communities in the USA.

## Additional files

Additional file 1: **Figure S1.** Stroke Ready Poster. (DOCX 674 kb)

Additional file 2: **Table S1.** Community Intervention Process and Implementation Measures. (DOCX 49 kb)

## Abbreviations

CAB: Community advisory board
CBPR: Community-based participatory research
CPT: Current procedural terminology
ED: Emergency department
FASt: Flint area study
GWTG: Get with Guidelines
ICER: Incremental cost-effectiveness ratio
IV tPA: Intravenous tissue plasminogen activator
PSAs: Public service announcements
QALYs: Quality adjusted life years
SID: State Inpatient Database
STYH: Speak to Your Health
TICD: Tailored implementation of chronic disease
