# Supplementary figures and images for: Stroke Ready: a multi-level program that combines implementation science and community-based participatory research approaches to increase acute stroke treatment: protocol for a stepped wedge trial
Source: Implement Sci. 2019 Mar 7;14:24. doi: 10.1186/s13012-019-0869-3 (PMC6407173; doi:10.1186/s13012-019-0869-3)

Supplemental Figure 1: Stroke Ready Poster


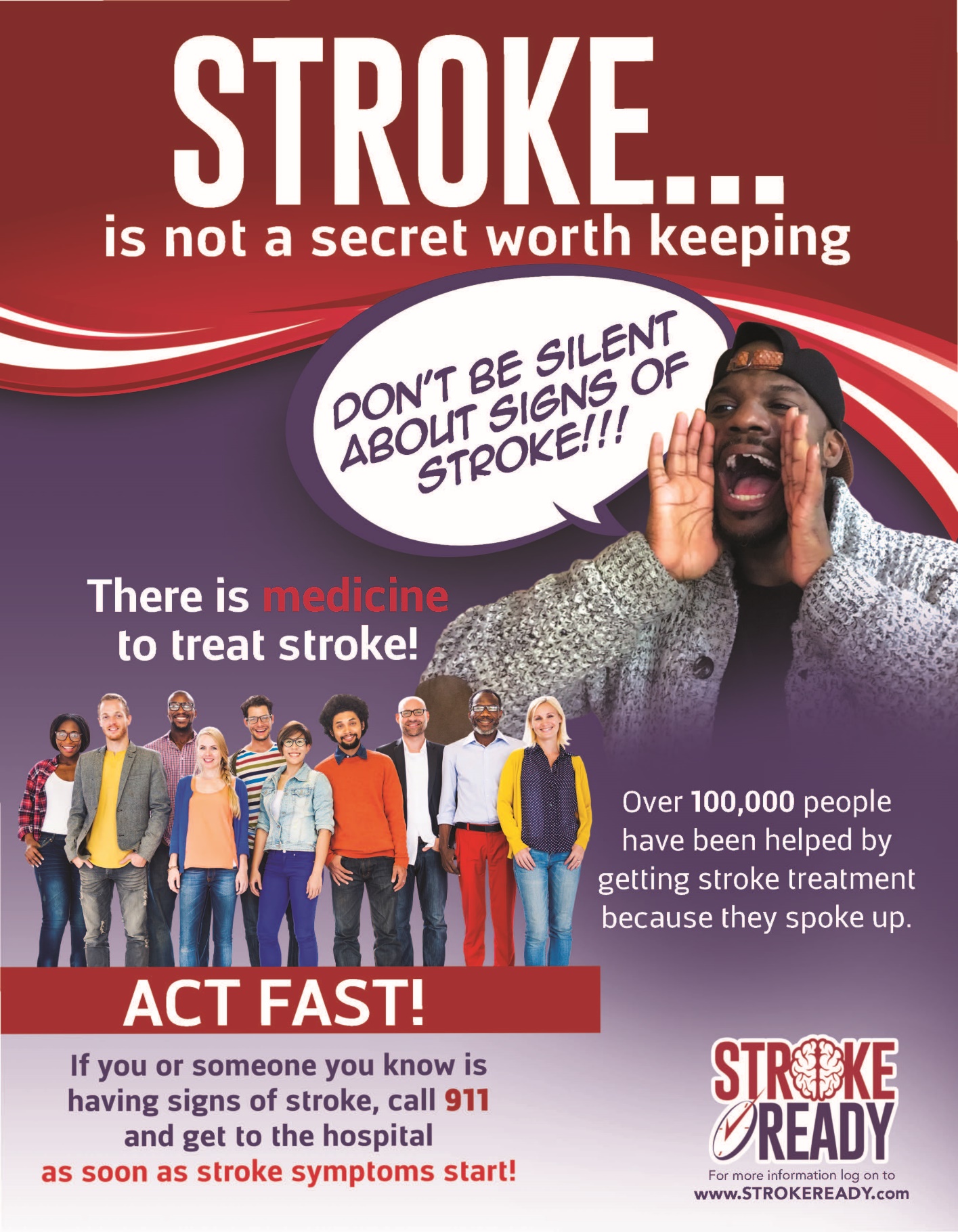

Supplement: Supplementary file 1 — Figure S1. Stroke Ready Poster. (DOCX 674 kb) [file 13012_2019_869_MOESM1_ESM.docx]
