# Supplementary material for: Stroke Ready: a multi-level program that combines implementation science and community-based participatory research approaches to increase acute stroke treatment: protocol for a stepped wedge trial
Source: Implement Sci. 2019 Mar 7;14:24. doi: 10.1186/s13012-019-0869-3 (PMC6407173; doi:10.1186/s13012-019-0869-3)
Supplement: Supplementary file 2 — Table S1. Community Intervention Process and Implementation Measures. (DOCX 49 kb) [file 13012_2019_869_MOESM2_ESM.docx]

| **Supplemental Table 1: Community Intervention Process and Implementation Measures** | | |
| --- | --- | --- |
| 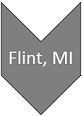 | - **Community Reach** | |
|  |  | **FASt Survey**  **Speak to Your Health Survey** |
|  | - **Broadcast media** | |
|  |  | **PSA uptake**  -Number of plays of PSAs on radio and TV per month  -Number of listening/viewing audience reached (by station) |
|  | - **Digital Media** | |
|  |  | **Website uptake**  -Number of visitors to Stroke Ready website  -Number of page views  -Number of hits to Stroke Ready website (from within Flint via IP address) |
|  |  | **Uptake: Music video**  -Number of views on website  -Number of YouTube views  -Number of views from within Flint |
|  |  | **Social media uptake (Facebook and Instagram)**  -Number of posts  -Number reached  -Number reached from within Flint  -Number of followers  -Number of site visits  -Number of page views  -Number of boosts  -Number reached by boosted content  -Number of boosted content views/engagement |
|  | | |
| 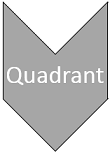 | - **Mailers** | |
|  |  | **Uptake: Mailers**  -Number sent to all residential households  -Number of community members who mention receiving mailer |
|  | - **Print Material** | |
|  |  | **Uptake: Posters**  -Number of sites  -Number of posters  -Number of posters remaining at sites after 3 months |
|  | - **Peer-Led Workshop** | |
|  |  | **Uptake: Workshops**  -Number of workshop sites  -Number of workshops held (each type) |
|  | - **Brief and Very Brief Sessions** | |
|  |  | **Uptake: Brief Sessions**  -Number of event sites  -Number of interventions (each type) |
|  | | |
| 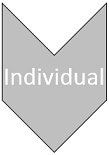 | - **Peer-Educator Led Workshop** | |
|  |  | -Number of workshop participants (number served)  -Number of workbooks distributed  -Workshop duration (by type)  -All intervention core components delivered  -Participant responsiveness and satisfaction  -Appropriateness of setting and context  -Implementation barriers or successes  -Intervention fidelity |
|  | - **Brief and Very Brief Sessions** | |
|  |  | -Number of participants receiving intervention  -Number of brochures/action plans distributed  -Duration of intervention (by type)  -All intervention core components delivered  -Participant responsiveness and satisfaction  -Appropriateness of setting and context  -Implementation barriers or successes  -Intervention fidelity |
|  | - **Cost-effectiveness** | |
|  | -Number of hours providing intervention vs. number of total hours worked by   peer educator | |
